# Comparative study on the protective effect of dexrazoxane and blueberry extract against doxorubicin-induced cardiotoxicity in rats

**DOI:** 10.1038/s41598-025-19853-3

**Published:** 2025-09-23

**Authors:** Nourhan Ahmed Shaker, Eman Abd El-Moneim Sharaf, Nesma Ali Ali Ghazal, Nagwa Mohamed Assem

**Affiliations:** Department of Biochemistry, Medical Research Institute, Alexandria University, 165 El-Horreya Avenue, El-Hadara, POB: 21561 Alexandria Egypt

**Keywords:** Cardiotoxicity, Doxorubicin, Dexrazoxane, Oxidative stress, Blueberry extract, Myocardium protection, Biochemistry, Molecular biology

## Abstract

The therapeutic efficacy of anthracycline antibiotic, doxorubicin (DOX), is hampered due to cardiotoxicity. The objective of the study was to explore the counteraction of blueberry (BB) extract and Dexrazoxane (DEX) in Dox-induced cardiotoxicity in Wistar rats. Screening of BB extract as well as DEX for protection the myocardium from Dox-induced oxidative stress was performed on seven groups (8 rats each): Control (normal diet for 14 days and IP injection of normal saline (10 ml/kg) on the 11th day), DOX control (normal diet for 14 days with a single DOX injection of 18 mg/kg on the 11th day), BB extract control (80 mg/kg), DEX (180 mg/kg on the 11th day), BB + DOX (80 mg/kg BB extract for 14 days with DOX on the 11th day, 18 mg/kg), DEX + DOX (180 mg/kg DEX 30 min before 18 mg/kg DOX on the 11th day), and a combined group BB + DOX + DEX. A significant increase in serum biomarkers cTnT, NT-proBNP, MPO and cardiac MDA, TOP II, and a significant decrease in GSH and SOD contents were observed in the cardiotoxic (DOX control) group. All these parameters were reversed significantly in all treated groups in comparison to cardiotoxic groups. The cardiotoxic group showed significant upregulation of miR-140-5p expression and significant downregulation of Sirt2 and Nrf2 expression reversed in all treated groups except miR-140-5p which showed unsignificant difference. The best ameliorative effect was observed in the combined group. The histopathological assessment of myocardial damage provided supportive evidence for the biochemical results obtained. In conclusion, the BB extract (80.0 mg/kg) can attenuate the DOX-induced oxidative stress, and it has the potential to be developed as an adjunct against DOX-induced cardiotoxicity in cancer patients who undergo anthracycline chemotherapy.

**Supplementary Information:**

The online version contains supplementary material available at 10.1038/s41598-025-19853-3.

## Introduction

Cardiotoxicity is a term used to describe damage and dysfunction in the heart. Cardiotoxicity can impair the heart’s ability to efficiently circulate blood throughout the body. Severe cardiotoxicity might potentially result in the development of cardiomyopathy. Cancer therapy often results in significant co-morbidity and death. Cardiotoxicity is a known side effect of various therapeutic interventions, such as radiotherapy, anthracycline**s** e.g. doxorubicin (Adriamycin^®^) and targeted therapy drug e.g. trastuzumab (Herceptin^®^)^1^.

Doxorubicin (DOX) is highly efficacious as a chemotherapeutic agent. Nevertheless, the practical use of this treatment is restricted due to the possibility of acute or chronic irreversible cumulative cardiotoxicity. It is promising that the growing body of evidence from fundamental research, preclinical studies, and clinical trials is shedding light on the underlying causes and molecular mechanisms of this disease^2^. This research has the potential to uncover new biomarkers for early detection and develop preventive strategies. Furthermore, recent data has linked DOX cardiotoxicity with genetic risk factors. The results obtained in this area of research will be advantageous for predicting the responsiveness of tumors to DOX treatment and the vulnerability of the population to DOX-induced heart damage^3,4^. Therefore, it is possible to develop and implement tailored strategies for individuals to obtain the most effectiveness in cancer treatment while minimizing any negative effects, based on the genetic variations specific to each patient^5^.

Dexrazoxane (DEX) is a powerful compound that acts as an intracellular iron chelating agent. It is derived from ethylenediaminetetraacetic acid (EDTA)^6^. DEX’s cardioprotective action is partially achieved by its ability to chelate iron and counteract the formation of reactive oxygen species (ROS) generated by DOX in cardiomyocytes^7^. DEX also protects the heart by reducing the levels of cardiac Topoisomerase IIβ (Top2β) protein through degradation mediated by the proteasome in cardiomyocytes^8^.

Blueberry (Vaccinium corymbosum L) is well acknowledged as a significant provider of anthocyanins^9^. The anthocyanins found in blueberries activated the cellular antioxidant system and prevented the expression of inflammatory genes. As a result, they provided protection against organ toxicity caused by oxidative stress or inflammation^10,11^. This protection has potential health benefits, such as safeguarding cardiovascular health, protecting the brain, preventing cancer, and managing diabetes. There is a hypothesis that extracts enhanced with blueberry anthocyanins extract (BAE) may improve the preventive effect of DEX against heart hypertrophy and damage^10^.

The objectives of this study were to compare the protective effects of Dexrazoxane (DEX) and blue berry extract against the cardiomyopathy induced by Doxorubicin (DOX) in rats and to investigate whether (DEX) alone or in combination with blueberry extract could prevent DOX-induced cardiac dysfunction and ameliorate DOX-induced mitochondrial damage in the heart.

## Materials and methods

### Animal Preparation and study design

Fifty-six male Wistar albino rats, aged 6–8 weeks and weighing 175 ± 25 g, were procured from the Medical Research Institute, Alexandria, Egypt. All animals were housed under controlled conditions with a 12:12-hour light-dark cycle, temperature maintained at 24 ± 2 °C, and relative humidity of 50 ± 10%. Animals had had ad libitum access to food and water. The study protocols were approved by the Institutional Animal Care and Use Committee (IACUC) of Alexandria University, Egypt (Approval No.: AU0122262212), conforming to the NIH guidelines for the care and use of laboratory animals (NIH Publications No. 8023, revised 1978) and the animal welfare regulations of Egypt.

### Euthanasia and tissue collection

At the end of the experimental period, rats were euthanized under deep anesthesia using isoflurane inhalation in a closed chamber (4–5% for induction, 1–2% for maintenance) via a precision vaporizer. Once deep unconsciousness was confirmed, euthanasia was performed by decapitation to obtain blood and heart tissues. Decapitation was chosen as it allows for rapid euthanasia while minimizing potential alterations in blood composition caused by anesthetic agents^12^. This method adheres to the American Veterinary Medical Association (AVMA) Guidelines for the Euthanasia of Animals (2020), which recognize decapitation under anesthesia as an acceptable method for preserving tissue integrity^13^.

### Types of treatment

1. Doxorubicin (Adricin Hikma product, 50 mg/25 ml) was in the form of liquid vial.
2. Dexrazoxane obtained from Biosynth Ltd (USA) was administered as a powder dissolved in diluted Dimethyl Sulfoxide (DMSO 5%)^14^.
3. Blueberry extract (Natural Factors Canada product) was in the form of liquid oil which was dissolved in olive oil.

### Induction of cardiotoxicity

Cardiotoxicity was induced by a single intraperitoneal (IP) injection of DOX at a dose of 18 mg/kg, dissolved in normal saline, administered on the 11th day of the study period^15^.

### Experimental grouping

Rats were randomized into seven groups, each consisting of eight animals, with the following treatments: Group I (Control) comprised normal rats that received a regular diet for 14 days and an IP injection of normal saline (1 ml/kg) on the 11th day. Group II (Cardiotoxic, DOX) consisted of rats that received a normal diet for 14 days and an IP injection of Doxorubicin (DOX) at a dose of 18 mg/kg on the 11th day to induce cardiotoxicity. Group III (BB Control) involved rats receiving daily oral supplementation of BB extract (80 mg/kg) for 14 days and an IP injection with a volume of normal saline equal to the DOX dose on the 11th day. Group IV (DEX Control) included rats on a normal diet for 14 days, receiving an IP injection of DEX at 180 mg/kg on the 11th day. Group V (BB + DOX) included rats that were given an oral dose of BB extract (80 mg/kg) daily for 14 days and an IP injection of DOX (18 mg/kg) on the 11th day. Group VI (DEX + DOX) involved rats receiving a single IP injection of DEX (180 mg/kg) 30 min before an IP injection of DOX (18 mg/kg) on the 11th day. Finally, Group VII (BB + DEX + DOX) consisted of rats receiving an oral dose of BB extract (80 mg/kg) daily for 14 days, an IP injection of DOX (18 mg/kg), and an IP injection of DEX (180 mg/kg) 30 min before DOX on the 11th day^15^.

### Sample collection and Preparation

Blood samples were collected 24 h post the last IP injection. Samples were centrifuged at 70,000 rpm for 10–12 min to separate serum, which was then stored at −80 °C until biochemical analysis. Whole heart tissues were excised, washed with saline, and divided for biochemical and histological analyses. One part was homogenized for biochemical assays and the other was fixed in 10% formalin for histological examination^16^.

### Biochemical assays

Biochemical parameters were assessed using various methods. Cardiac Troponin T (cTnT) and Topoisomerase IIβ (Top2β) were quantified via ELISA. N-Terminal Pro-BNP (NT-proBNP) levels were measured with a rat-specific sandwich ELISA. Myeloperoxidase (MPO) activity was evaluated using a colorimetric assay based on taurine oxidation. Lipid peroxidation, indicated by malondialdehyde (MDA) levels, was assessed using the Thiobarbituric Acid Reactive Substances (TBARS) assay. Reduced Glutathione (GSH) was determined colorimetrically with Dinitrobenzoic acid, while Superoxide Dismutase (SOD) activity was measured by its inhibition of pyrogallol autoxidation.

### Gene expression analysis

Gene expressions of miR-140-5p, Nrf2, and Sirt2 were analyzed using quantitative real-time PCR (qRT-PCR). Total RNA was isolated using the miRNeasy Mini Kit **(Qiagen**,** Germany**,** Cat. No. 217004)**, converted to cDNA, and amplified using SYBR Green chemistry.

### Histopathological examination

Heart sections were stained with Hematoxylin and Eosin (H&E) for structural examination under a light microscope^17^.

### Statistical analysis

Data was analyzed using SPSS software **(version 20.0**,** Chicago**,** IL**,** USA).** Normality of distribution was verified using the Kolmogorov-Smirnov test. Differences among groups were evaluated using one-way ANOVA followed by Tukey’s post hoc test. Correlations were assessed using Pearson’s coefficient^18^. Statistical significance was set at *p* < 0.05.

## Results

### Serum biomarkers of cardiac injury and inflammation

The impacts on serum levels of cardiac troponin-T (cTnT), N-terminal pro-brain natriuretic peptide (NT-proBNP), and Myeloperoxidase (MPO) are discussed and visually represented in Table 1. In the DOX group, there was a significant increase in cTnT, NT-proBNP, and MPO levels compared to the Control group, indicating elevated cardiac stress. In the BB Control group, cTnT levels significantly decreased, but NT-proBNP levels remained unchanged, while MPO levels increased compared to the Control group. The DEX group showed no significant change in cTnT levels but had significant reductions in both NT-proBNP and MPO levels compared to the Control group, suggesting a potential protective effect. Among the treated groups, all demonstrated significant reductions in cTnT, NT-proBNP, and MPO levels compared to the DOX group, with the most pronounced improvements observed in the group treated with both BB and DEX.

**Table 1:** Serum levels of cTnT, NT-proBNP and MPO in the studied groups.

Parameter	Control	DOX	BB	DEX	DOX + BB	DOX + DEX	BB + DOX + DEX
cTnT (pg/mL)	24.17^ed^ ± 2.11	47.94^a^ ± 1.64	21.56^gf^ ± 1.02	22.88^ef^ ± 2.03	33.69^b^ ± 1.29	27.84^c^ ± 1.33	25.55^d^ ± 0.97
NT-pro BNP (pg/mL)	68.56^e^ ± 2.44	368.50^a^ ± 2.45	67.19^ef^ ± 1.33	65.88^fg^ ± 1.75	146.63^b^ ± 2.08	114.75^c^ ± 2.36	96.06^d^ ± 2.88
MPO (U/ml)	22.01^f^ ± 2.01	48.06^a^ ± 2.70	25.30^d^ ± 1.27	20.31^g^ ± 1.51	29.23^b^ ± 0.44	27.13^c^ ± 0.87	24.64^de^ ± 0.85

Data are expressed as mean ± SD (*n* = 8). Groups are identified in the header row as follows: Control (g), DOX (f), BB (e), DEX (d), DOX + BB (c), DOX + DEX (b), and BB + DOX + DEX (a). Within each parameter row, superscript letters denote statistical significance: values sharing the same letter are not significantly different, while values with different letters differ significantly (*p* < 0.05, one-way ANOVA with Tukey’s post hoc test). A value carrying two letters (e.g., ef) indicates no significant difference from either group e or group f.

### Oxidative stress and antioxidant parameters in cardiac tissues

Changes in malondialdehyde (MDA), reduced glutathione (GSH), and superoxide dismutase (SOD) levels in cardiac tissues are summarized in Table 2. The DOX group showed a significant increase in MDA content, indicating higher lipid peroxidation, along with a corresponding decrease in GSH and SOD levels compared to the Control group, reflecting increased oxidative stress. In contrast, the BB and DEX groups exhibited no significant change in MDA content compared to the Control group. However, the DEX group showed a decrease in GSH content and an increase in SOD content. In the treated groups, there was a significant reduction in MDA content and increases in GSH and SOD levels compared to the DOX group, with the most favorable outcomes observed in the combination treatment group (BB + DEX).

**Table 2:** Cardiac contents of MDA, GSH and SOD in the studied groups.

Parameter	Control	DOX	BB	DEX	DOX + BB	DOX + DEX	BB + DOX + DEX
MDA (nmol/gm tissue)	3.06^g^ ± 0.27	29.99^a^ ± 1.80	3.41^gfe^ ± 0.48	3.86^ge^ ± 0.39	12.39^b^ ± 0.83	10.21^c^ ± 0.55	7.24^d^ ± 0.55
GSH (µmol/gm tissue)	2.10^ab^ ± 0.15	0.37^g^ ± 0.10	2.16^a^ ± 0.16	1.95^c^ ± 0.10	1.45^f^ ± 0.11	1.71^e^ ± 0.07	1.94^cd^ ± 0.09
SOD (U/mg protein)	31.93^bc^ ± 1.85	6.83^g^ ± 1.38	33.30^ab^ ± 0.90	33.81^a^ ± 2.18	16.50^f^ ± 0.97	22.66^e^ ± 1.03	26.82^d^ ± 1.00

Data are expressed as mean ± SD (*n* = 8). Groups are identified in the header row as follows: Control (g), DOX (f), BB (e), DEX (d), DOX + BB (c), DOX + DEX (b), and BB + DOX + DEX (a). Within each parameter row, superscript letters denote statistical significance: values sharing the same letter are not significantly different, while values with different letters differ significantly (*p* < 0.05, one-way ANOVA with Tukey’s post hoc test). A value carrying two letters (e.g., ef) indicates no significant difference from either group e or group f.

### Cardiac levels of topoisomerase IIβ (TOP IIβ)

**Fig. 1:**
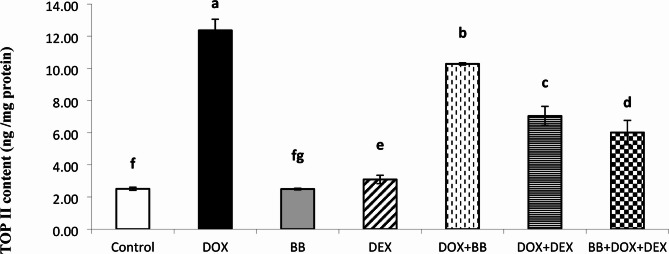
Cardiac Topoisomerase IIβ (TOP IIβ) protein levels (ng/mg protein) across all studied groups. A significant increase was observed in the DOX group compared to the control, while combination treatment with BB and DEX markedly reduced TOP IIβ levels.

The expression levels of TOP IIβ are presented in Fig. 1. Both the DOX and DEX groups showed increased levels of TOP IIβ compared to the Control group, whereas the combination treatment groups, especially BB + DEX, displayed the most effective reduction in TOP IIβ levels relative to the DOX group.

### Molecular gene expression studies

**Fig. 2:**
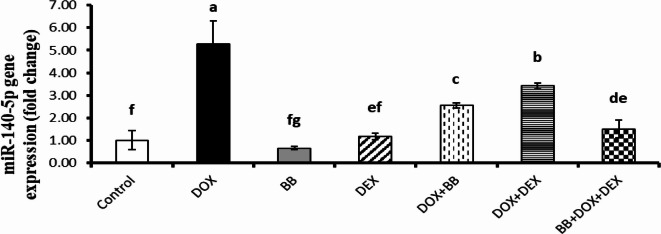
Fold change in miR-140-5p gene expression in rat cardiac tissue. DOX administration significantly upregulated miR-140-5p, while treatment with BB, DEX, or their combination mitigated this elevation.

**Fig. 3:**
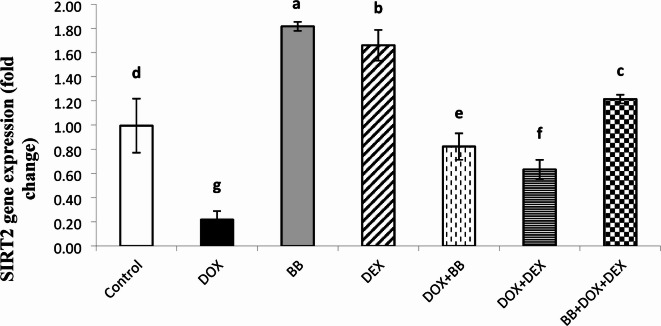
Fold change in Sirtuin 2 (SIRT2) mRNA expression in cardiac tissue. DOX treatment suppressed SIRT2 expression, whereas treatment with BB, DEX, or both led to significant upregulation.

**Fig. 4:**
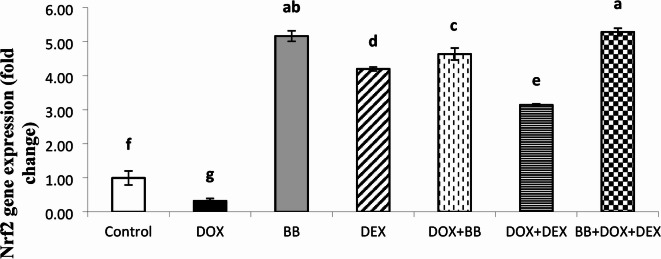
Relative Nuclear factor erythroid 2–related factor 2 (Nrf2) expression levels in the heart tissue of treated rats. Nrf2 expression was downregulated in the DOX group but restored by BB and DEX treatments. Highest expression was observed in the BB + DEX + DOX group.

The expression levels of miR-140-5p, Sirt2, and Nrf2 were evaluated and are summarized in Figs. 2, 3 and 4. In the DOX group, there was a significant upregulation of miR-140-5p and a downregulation of Sirt2 and Nrf2 expressions, indicating altered gene regulation associated with stress responses. In the BB and DEX groups, there was no significant change in miR-140-5p levels, but both groups showed significant upregulation of Sirt2 and Nrf2 expressions, suggesting enhanced protective mechanisms. In the treated groups, there was a notable downregulation of miR-140-5p and upregulation of Sirt2 and Nrf2 expressions, with the most pronounced effects observed in the combination treatment group, highlighting the enhanced benefits of the combined therapy.

### Correlation studies

Pearson correlation analysis revealed significant relationships among the studied biomarkers. Sirt2 expression was positively correlated with GSH and SOD levels, indicating its role in enhancing antioxidant defenses, and negatively correlated with MPO, MDA contents, and NT-proBNP levels, suggesting a protective effect against oxidative stress and cardiac damage. TOP IIβ content showed strong positive correlations with MDA, MPO, cTnT, and NT-proBNP levels, indicating its association with markers of cardiac damage. Additionally, miR-140-5p expression displayed positive correlations with MPO, MDA, and NT-proBNP levels, suggesting its involvement in cardiac stress responses A detailed correlation analysis is provided in Supplementary Table S1. (Supplementary file 1).

### Histopathological examination

**Fig. 5:**
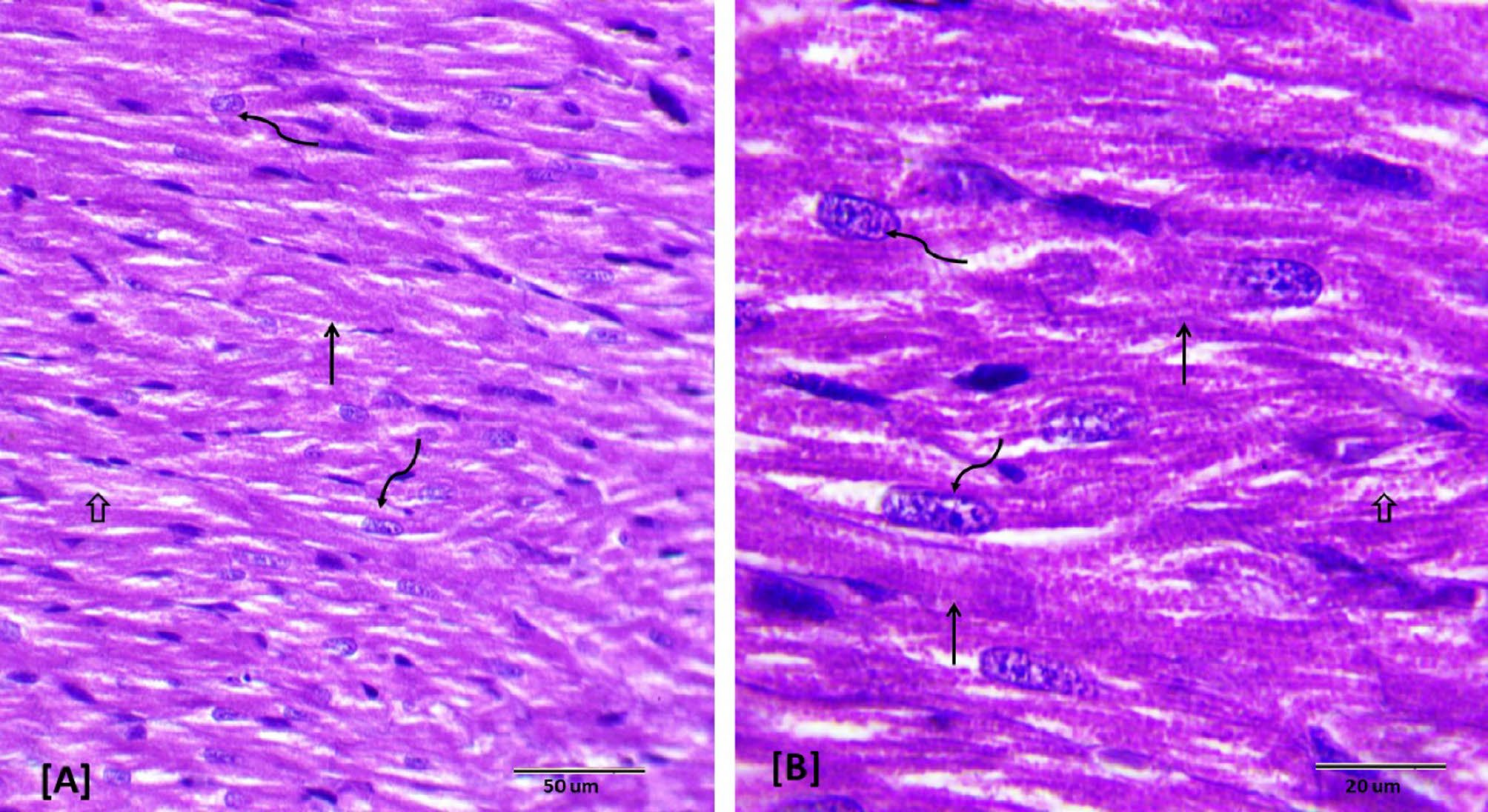
Descriptive photomicrographs of heart section from control rat. Showing histological pattern of branched cardiac muscles, longitudinally oriented normal cardiomyocytes (↑) with eosinophilic cytoplasm and oval nuclei with granular chromatin (wavy arrows). Note connective tissue between muscle fibers (↑). (H&E- Bar 50 μm and 20 μm), (**A** with magnification of 400 x and **B** with magnification of 1000 x).

**Fig. 6:**
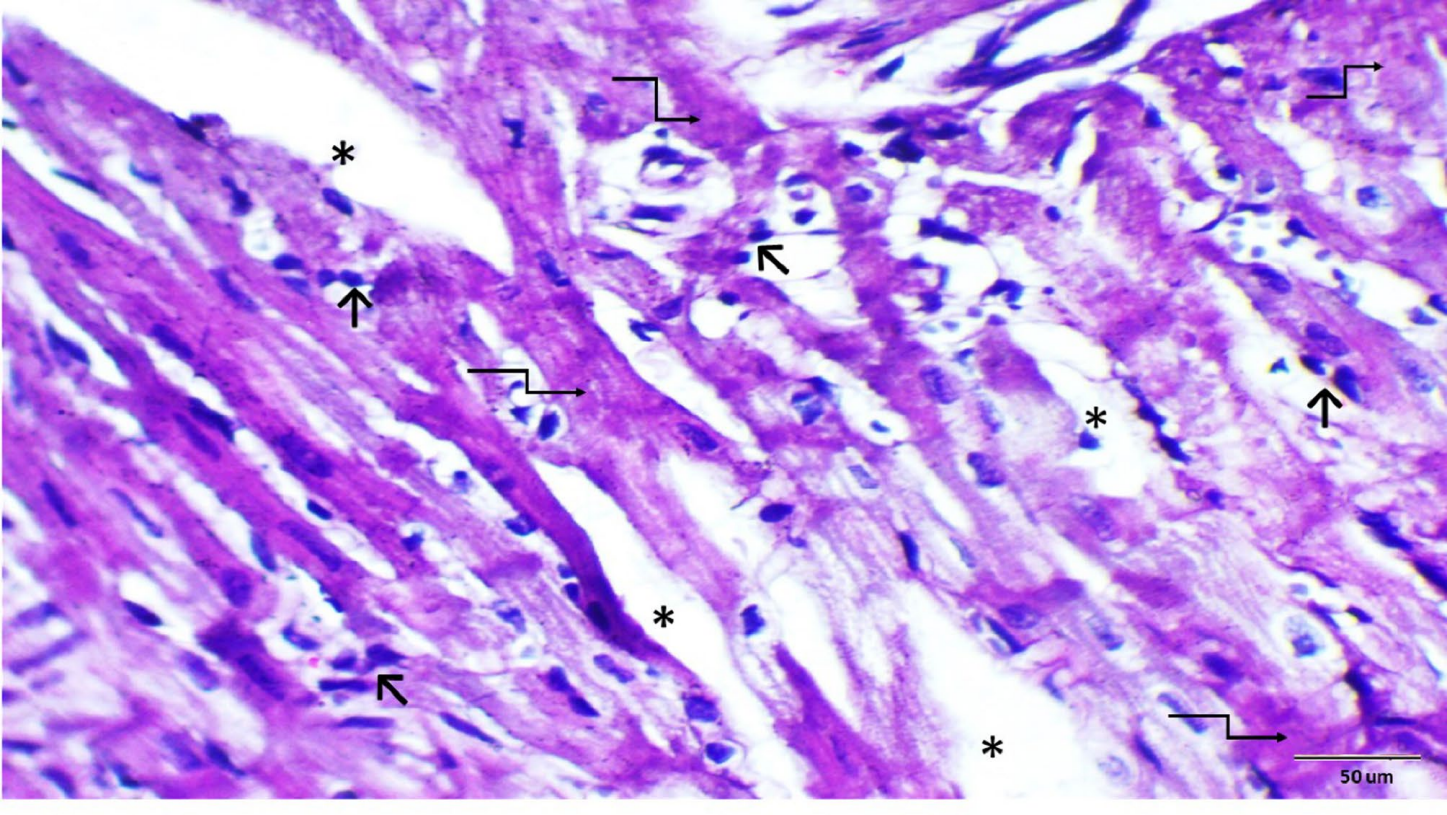
Photomicrograph of rat heart section from DOX-treated group. Showing myocardial damage illustrated as distorted myocardium with impairment of cellular details, area of necrosis (elbow arrow), widened spaces (*) between the cardiac muscle fibers and inflammatory cell infiltration (short arrow). (H&E- Bar 50 μm), (magnification = 400 x).

**Fig. 7:**
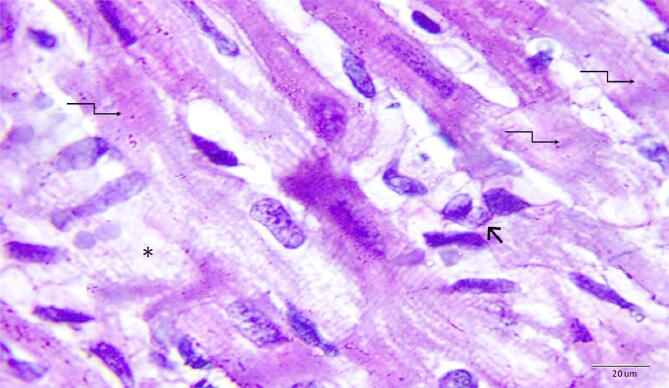
Higher magnification of the previous (Fig. 9). Showing representative area of necrosis (elbow arrow) and inflammatory cell infiltration (short arrow). (H&E- Bar 20 μm), (magnification = 1000 x).

**Fig. 8:**
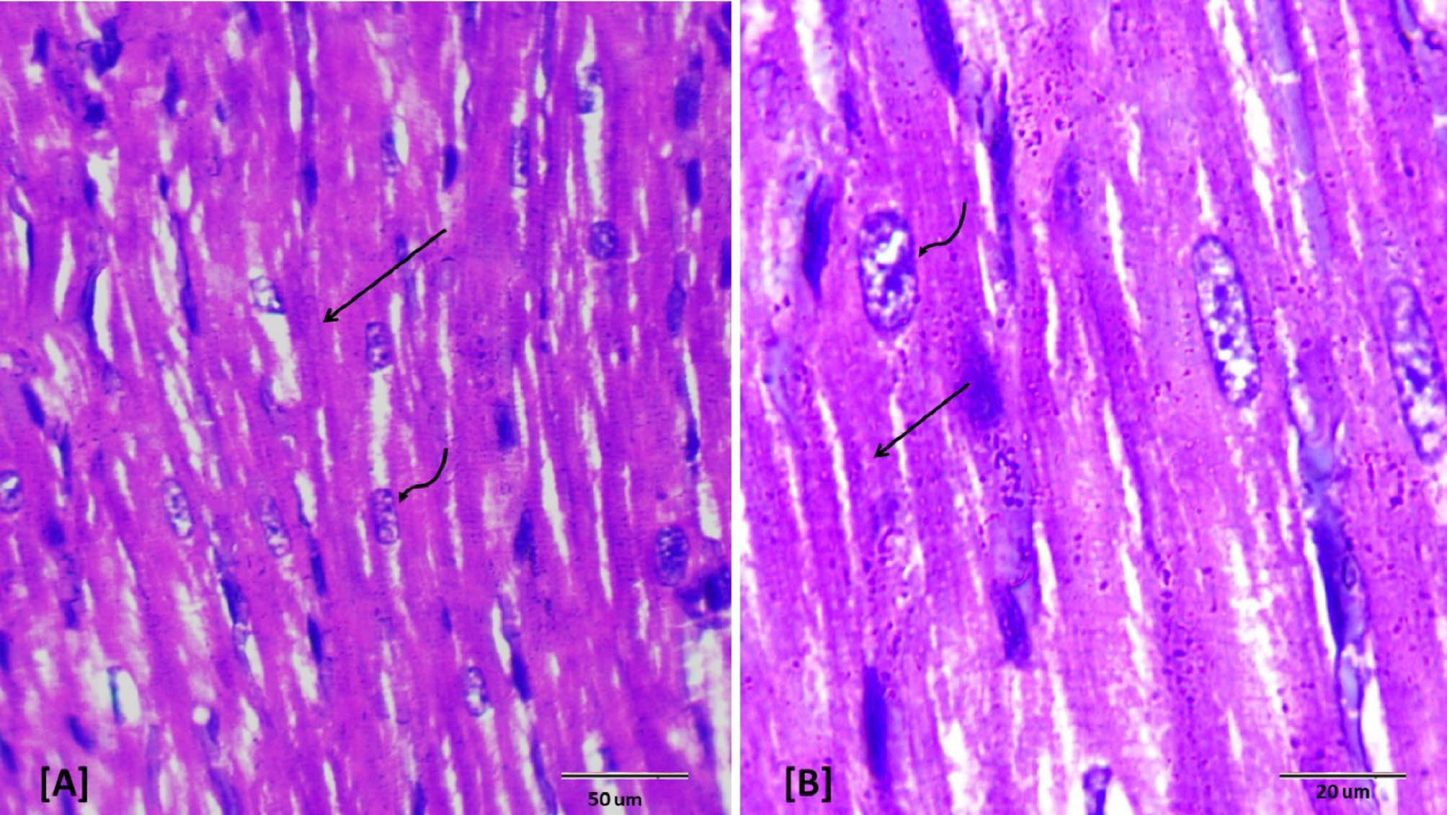
Photomicrographs of rat heart section from dexrazoxane treated group. Showing normal cardiomyocytes (↑) with eosinophilic cytoplasm and oval nuclei with granular chromatin (wavy arrows) as observed in control sections. (H&E- Bar 50 μm and 20 μm), (**A** with magnification of 400 x and **B** with magnification of 1000 x).

**Fig. 9:**
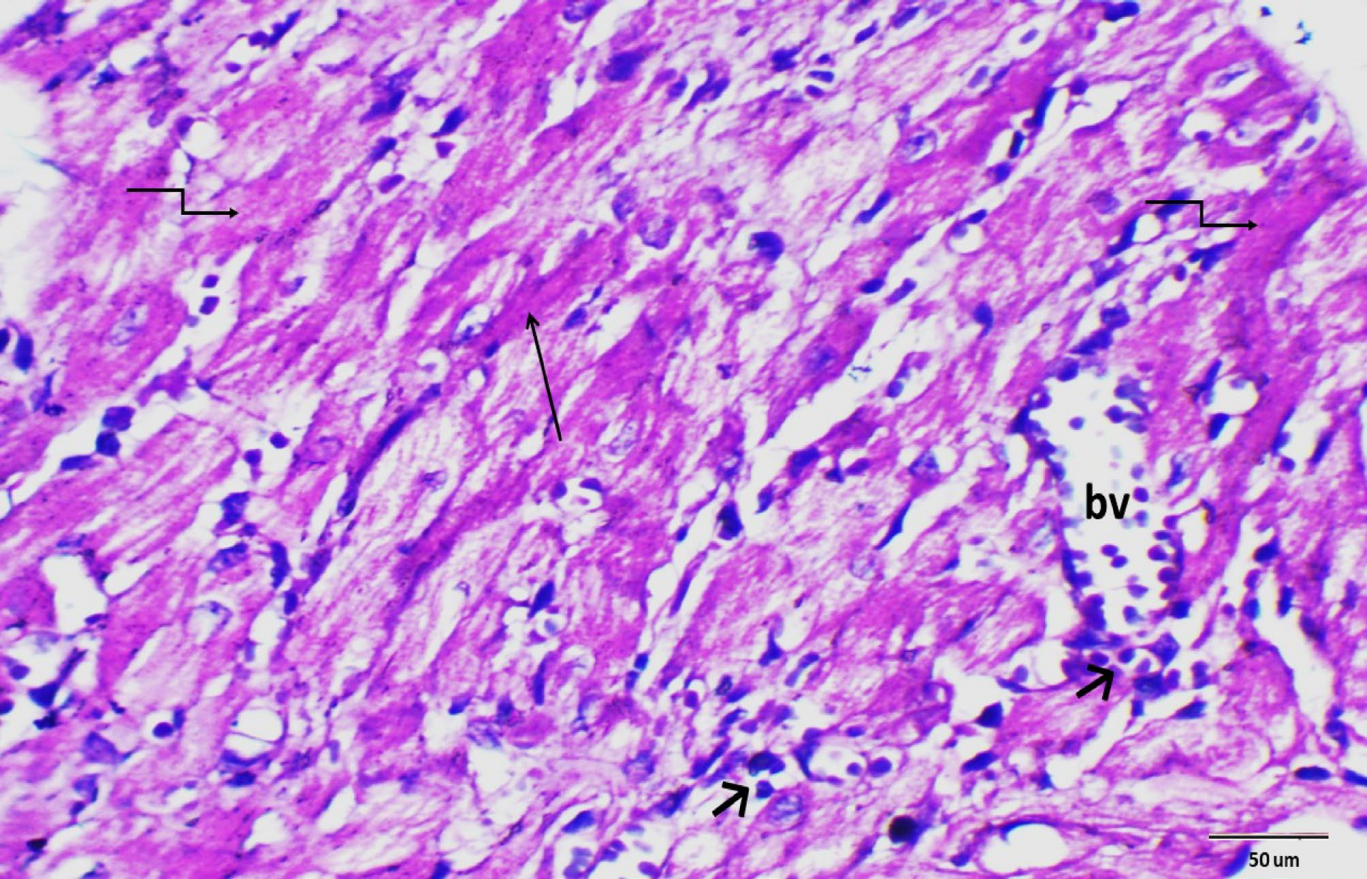
Photomicrograph of rat heart section from group treated with BB in combination with DOX. Showing distorted cardiac muscles (↑), necrosis (elbow arrow), inflammatory cell infiltration (short arrow) and congested blood vessel (bv). (H&E- Bar 50 μm), (magnification = 400 x).

**Fig. 10:**
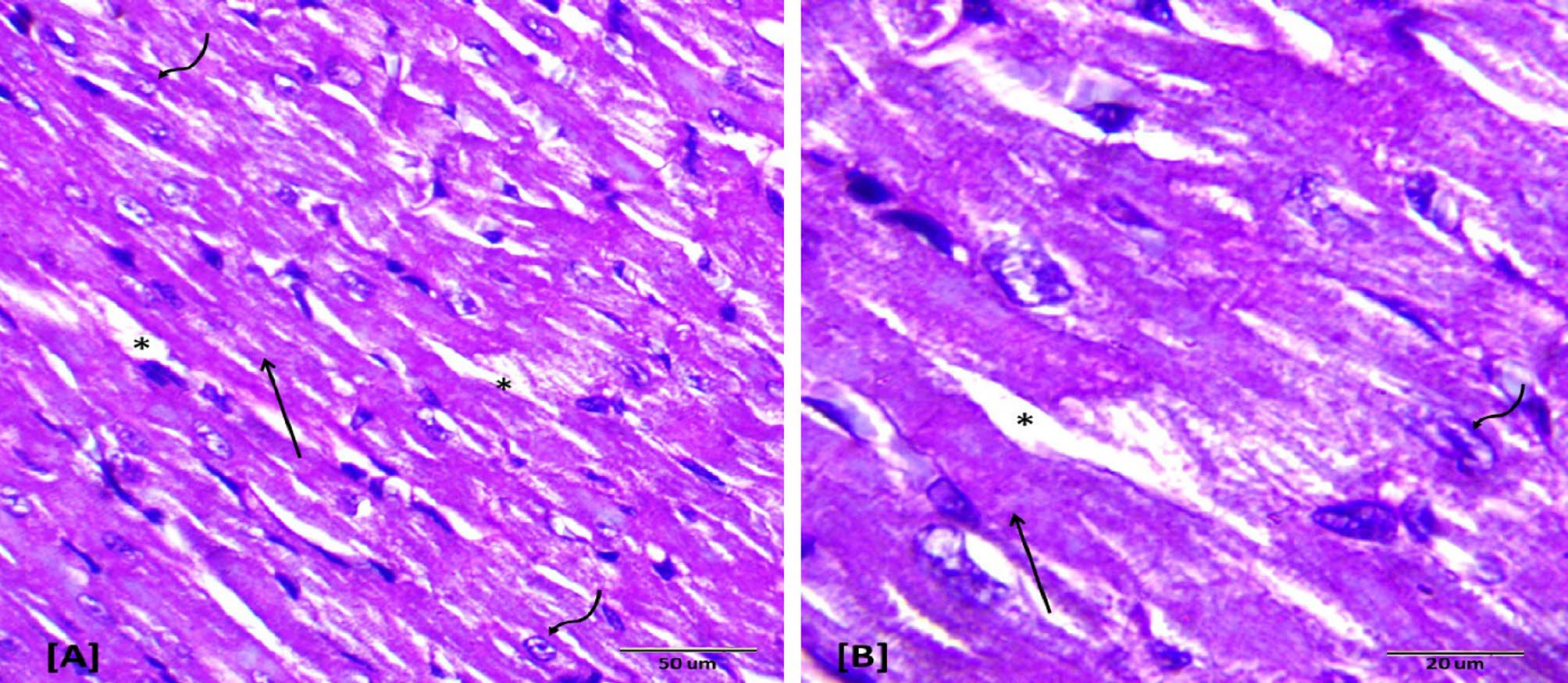
Photomicrographs of rat heart section from group (VI) treated with dexrazoxane plus DOX. Showing moderate restored cardiomyocytes architecture (↑) with no cellular infiltration. Note slightly widened spaces between the cardiac muscle fibers (*). (H&E- Bar 50 μm and 20 μm), (**A** with magnification of 400 x and **B** with magnification of 1000 x).

**Fig. 11:**
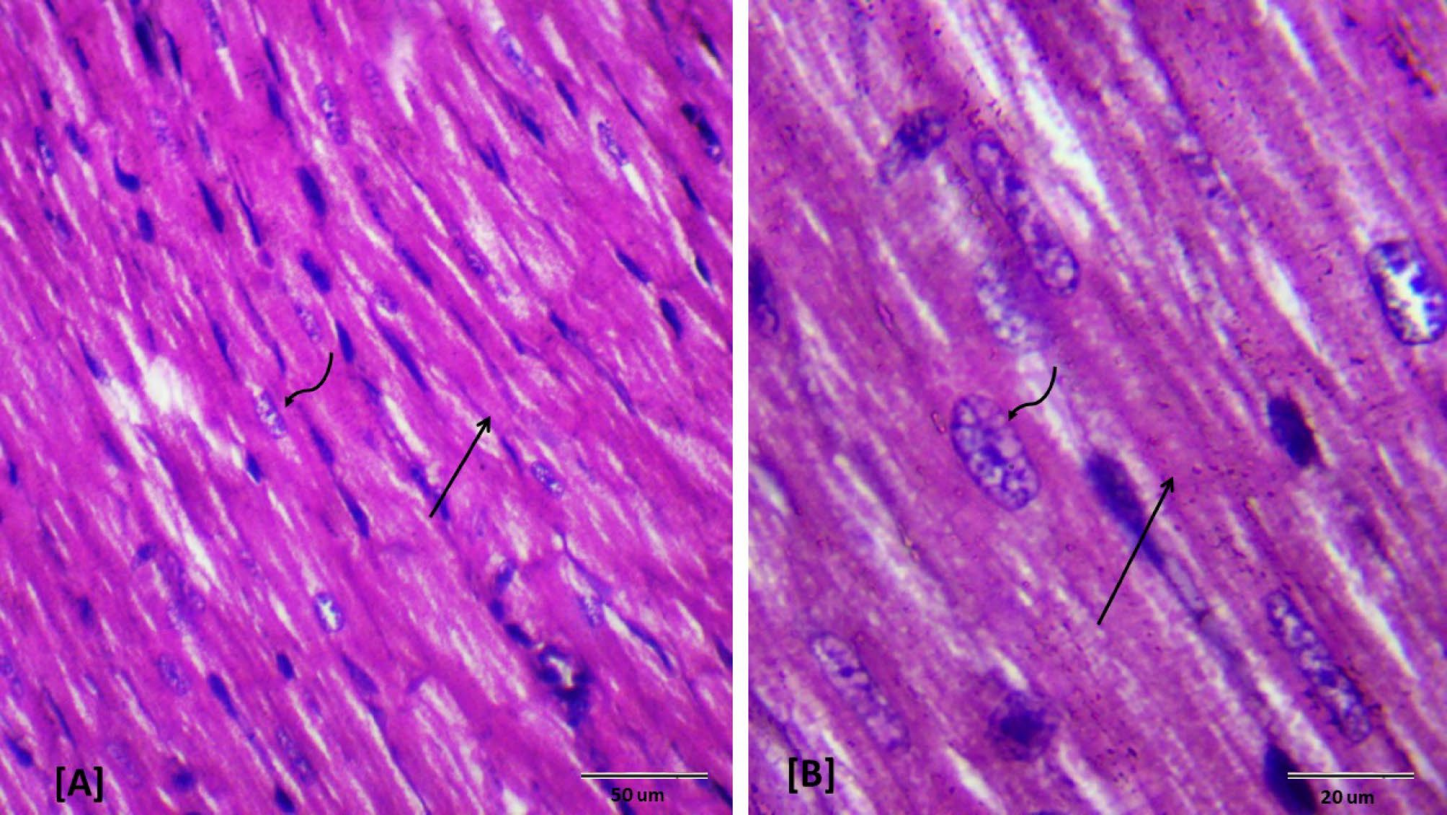
Photomicrographs of rat heart section from group (VII) treated with dexrazoxane and DOX after oral administration of BB. Showing amelioration of cardiac tissue; normal longitudinally oriented cardiomyocytes construction (↑) as seen in control group, (H&E- Bar 50 μm and 20 μm), **A** with magnification of 400 x and **B** with magnification of 1000 x).

Histopathological evaluations provided visual confirmation of biochemical data, indicating the protective effects of BB and DEX against DOX-induced cardiac damage, as detailed in Figs. 5, 6, 7, 8, 9, 10 and 11. In the Control group, normal cardiac morphology was observed, serving as a baseline for comparison. The DOX group showed notable cardiac damage characterized by necrosis and inflammation, highlighting the extent of cardiac injury. In contrast, the BB and DEX groups exhibited preservation of normal cardiac architecture, indicating their cardioprotective effects. Among the combination treatments, there was significant amelioration of DOX-induced damage, with the most pronounced improvements observed in the BB + DEX group, underscoring the enhanced efficacy of the combined therapy. These results collectively underscore the cardioprotective effects of BB and DEX, either alone or in combination, against DOX-induced cardiotoxicity, mediated through modulations of oxidative stress, inflammation, and cardiac injury biomarkers.

To reduce subjectivity, a semi-quantitative scoring system (0–3 scale) was applied to assess myocardial degeneration, necrosis, inflammatory infiltration, and fibrosis. Results are shown **in** Table 3, which confirm that the combination of BB and DEX provided the greatest reduction in histopathological damage, consistent with biochemical findings.

**Table 3:** Semi-quantitative histopathological scoring of cardiac injury.

Group	Myocardial degeneration	Necrosis	Inflammatory infiltration	Fibrosis
Control	0	0	0	0
DOX	3	3	3	2
BB	0	0	0	0
DEX	0	0	0	0
DOX + BB	2	2	2	1
DOX + DEX	1	1	1	0
BB + DOX + DEX	0–1	0–1	0–1	0

Scoring system: 0 = absent, 1 = mild, 2 = moderate, 3 = severe. Evaluation performed by blinded pathologist.

## Discussion

Oncological cardiology, an emerging discipline, intersects oncology, cardiovascular medicine, imaging, laboratory sciences, and other fields. With the continuous in-depth research on antitumor drugs, various chemotherapeutic and new targeted drugs are being widely used in clinical settings. However, their adverse effects cannot be ignored, as they may be major causes of death^19^. Cardiotoxicity resulting from chemotherapy drugs and immune checkpoint inhibitors (ICIs) primarily manifests as various types of cardiac dysfunction, heart failure (HF), and myocarditis, potentially associated with high morbidity and mortality^20^.

Anthracyclines like doxorubicin, epirubicin, daunorubicin, and idarubicin are pivotal in treating various cancers, including both solid tumors and hematologic malignancies. However, their clinical utility is significantly constrained by dose-dependent cardiotoxicity, which presents a major challenge in oncology^5,21,22^. The risk of cardiotoxicity persists even at cumulative doses of doxorubicin below the traditionally cited threshold of 400 mg/m², suggesting a nuanced understanding of “safe” dosage is necessary^22–24^.

The primary pathophysiological mechanism of doxorubicin-induced cardiotoxicity involves the overproduction of reactive oxygen species (ROS), which leads to oxidative stress and cellular damage^25^. This oxidative stress is exacerbated by the affinity of doxorubicin for cardiolipin, a component of the inner mitochondrial membrane, disrupting mitochondrial function and enhancing ROS production^26,27^.

Given the critical role of oxidative stress in doxorubicin-induced cardiotoxicity, there is an emerging interest in integrating antioxidant therapies within treatment protocols. Currently, dexrazoxane is the only FDA-approved drug to mitigate this side effect, but its use is limited by potential adverse effects and contraindications in certain patient populations^21^. This limitation has spurred interest in natural antioxidants, such as those found in blueberries, which are rich in anthocyanins, flavonoids, and phenolic acids known for their robust antioxidant properties^28^.

Our findings suggest that blueberry extract can significantly reduce cardiac biomarkers such as cTnT and NT-proBNP, indicators of cardiac injury, in doxorubicin-treated rats. This suggests a protective effect against cardiotoxicity, likely due to the high polyphenol content of the extract, which mitigates oxidative damage^29^. Furthermore, the extract was observed to enhance the activities of endogenous antioxidants like glutathione and superoxide dismutase, essential in counteracting the oxidative stress induced by doxorubicin treatment.

The results from this study are consistent with prior research indicating that natural extracts rich in polyphenols can significantly reduce oxidative stress markers such as malondialdehyde, a byproduct of lipid peroxidation, and improve the antioxidative defense systems in cardiac tissues^30–34^. These findings not only reinforce the concept of using natural antioxidants as adjuvants in chemotherapeutic regimens but also highlight their potential in reducing the side effects associated with conventional cancer therapies.

Inflammation also plays a crucial role in doxorubicin-induced cardiotoxicity, with increased activation of NF-κB signaling and subsequent cytokine production. Interestingly, our study demonstrated that blueberry extract not only reduces oxidative stress but also mitigates inflammatory responses, as evidenced by the decreased myeloperoxidase (MPO) activity and inflammatory cytokine levels^35^. The blueberry group’s reduction in MDA and MPO was linked to NF-κB modulation, while dexrazoxane protection was attributed to iron chelation and Top2β inhibition.

The complex interplay of oxidative stress, inflammation, and apoptosis in doxorubicin cardiotoxicity underscores the potential of multifunctional therapeutic agents like blueberry extract that can simultaneously address these pathological mechanisms. This holistic approach could significantly enhance the safety and efficacy of anthracycline-based chemotherapy, providing a compelling case for further research into natural antioxidants as cardioprotective agents in oncology.

Topoisomerase IIβ (Top IIβ) is a key factor in DOX-induced cardiotoxicity. Zhang et al. showed in a mouse model that deleting the Top IIβ gene in the heart reduced double strand breaks, apoptosis, and variations in gene expression related to mitochondrial production. This gene deletion prevented heart failure from DOX, highlighting Top IIβ’s crucial role. Dexrazoxane reduces DOX-induced cardiotoxicity by attaching to the ATPase domain of Top II and breaking down Top IIβ^36^. The study found significant elevation of Top II in the DOX and Dexrazoxane groups, with the lowest levels in cardiotoxic rats treated with a combination of BB and DEX. In line with the present study, the cardiotoxic group (DOX group) and the Dexrazoxane group showed significant elevation of TOP II while the lowest levels of TOP II content were observed significantly in the cardiotoxic rats treated with the combination of BB and DEX followed by the cardiotoxic rats treated with DEX alone.

DOX elevates miR-140-5p and reduces its targets, Nrf2 and SIRT2, increasing oxidative stress in the heart and causing cardiac damage. SIRT2 regulates oxidative stress and activates antioxidant genes via miRNA. Zhao et al. found that reducing SIRT2 and Nrf2 contributes to DOX-induced oxidative damage in H9c2 cells. In mice, DOX treatment correlated with increased miR-140-5p and decreased SIRT2. Blocking miR-140-5p reduces oxidative damage by restoring Nrf2, SIRT2, and their antioxidant genes. Dioscin injection in rats lowers miR-140-5p and increases SIRT2 and Nrf2, mitigating DOX-induced oxidative stress and cardiac damage^37^.

In the present study, all treated groups showed significant downregulation of miR-140-5p expression in comparison to cardiotoxic group (DOX group). While there was significant upregulation of SIRT2 and Nrf2 expression as compared to cardiotoxic group (DOX group).

These biochemical findings were corroborated by histopathological analyses of cardiomyocytes. Histopathological assessments of DOX group revealed cardiac damage characterized by intermuscular edema, loss of myofibrils, infiltration by inflammatory cells, vacuolization, and degeneration of cardiomyocytes. Moreover, pre-treatment with dexrazoxane mitigated the histological changes in cardiomyocytes caused by DOX, in comparison to samples treated solely with DOX^38^. This preservation of cardiomyocyte integrity contributed to the reduced serum levels of cardiac biomarkers noted in this study.

Histopathological evidence of cellular necrosis is a gold standard for identifying DOX-induced cardiotoxicity in rodents^39,40^. In our study, DOX-treated rats exhibited significant myocardial damage, including necrosis, inflammatory cell infiltration, and distorted myocardial damage. These findings align with Sun et al., who reported myocardial fibrosis and necrosis following DOX administration^41^.

Rats treated with either BB or DEX alone showed preservation of normal myocardial architecture, indicating their independent cardioprotective effects. This is consistent with findings by Sun et al., who reported similar outcomes using scutellarin due to its antioxidant properties^41^. However, in the BB + DOX group, only partial protection was observed—characterized by reduced but persistent signs of myocardial necrosis and mild inflammatory infiltration. This aligns with Zhang et al., who reported that oxymatrine alone was insufficient to fully prevent DOX-induced damage^42^. These findings suggest that while BB extract demonstrates protective potential, it may not be adequate as a standalone therapy against DOX-induced cardiotoxicity, thus supporting the enhanced efficacy seen when combined with DEX.

The combination of DEX and DOX showed moderate restoration of cardiomyocyte architecture, with fewer signs of damage^42^. Iqbal et al., similarly, found that telmisartan (an angiotensin II receptor blocker (ARB)) reduced DOX-induced histological changes. The most notable result was from the group treated with both DEX and BB along with DOX, which displayed nearly normal histological patterns, suggesting an enhanced cardioprotective effect^43^.

These results highlight the potential of DEX and BB, particularly in combination, to mitigate DOX-induced cardiotoxicity and restore normal myocardial structure.

Correlation studies confirmed with biochemical, molecular, and histopathological investigations in rats’ sera and cardiac tissues.

### Study limitations and future directions

This study has some limitations. Protein expression (Western blot/IHC) and total antioxidant capacity (TAC) were not assessed, which may have provided further mechanistic insight. In addition, functional assessments such as echocardiography were not performed, limiting translational value. Future studies should include protein validation, vehicle controls, and clinical evaluation to confirm the adjunctive cardioprotective potential of blueberry extract.

## Conclusion

Dexrazoxane and blueberry extract effectively reduce the cardiotoxic effects of doxorubicin, suggesting their potential inclusion in chemotherapy protocols to enhance patient safety and treatment efficacy.

## Supplementary Information

Below is the link to the electronic supplementary material.

Supplementary Material 1

## Data Availability

The data that supports the findings of this study are available from the corresponding author, upon reasonable request.

## Abbreviations

ANOVA: Analysis of Variance
AVMA: American Veterinary Medical Association
BAE: Blueberry anthocyanins extract
BB: Blueberry
cTnT: Cardiac Troponin T
DEX: Dexrazoxane
DOX: Doxorubicin
DMSO: Dimethyl Sulfoxide
EDTA: Ethylenediaminetetraacetic acid
ELISA: Enzyme-Linked Immunosorbent Assay
GSH: Reduced Glutathione
H&E: Hematoxylin and Eosin
IACUC: Institutional Animal Care and Use Committee
IP: Intraperitoneal
MDA: Malondialdehyde
MPO: Myeloperoxidase
mRNA: Messenger RNA
NF-κB: Nuclear factor kappa-light-chain-enhancer of activated B cells
NIH: National Institutes of Health
NT-proBNP: N-Terminal Pro-BNP (Brain natriuretic peptide)
qRT-PCR: Quantitative real-time PCR
ROS: Reactive oxygen species
SOD: Superoxide Dismutase
SPSS: Statistical Package for the Social Sciences
TBARS: Thiobarbituric Acid Reactive Substances
Top2β: Topoisomerase IIβ
